# Culture and characterization of various porcine integumentary-connective tissue-derived mesenchymal stromal cells to facilitate tissue adhesion to percutaneous metal implants

**DOI:** 10.1186/s13287-021-02666-2

**Published:** 2021-12-18

**Authors:** Devaveena Dey, Nicholas G. Fischer, Andrea H. Dragon, Elsa Ronzier, Isha Mutreja, David T. Danielson, Cole J. Homer, Jonathan A. Forsberg, Joan E. Bechtold, Conrado Aparicio, Thomas A. Davis

**Affiliations:** 1 Department of Surgery, Uniformed Services University of the Health Sciences, 4301 Jones Bridge Road, Bethesda, MD 20814 USA; 2 Henry M Jackson Foundation for Advancement of Military Medicine, Bethesda, USA; 3 Department of Restorative Sciences and MDRCBB-Minnesota Dental Research Center for Biomaterials and Biomechanics, University of Minnesota, Minneapolis, MN USA; 4 Hennepin Healthcare Research Institute, Minneapolis, MN USA; 5 Department of Orthopedic Surgery, University of Minnesota, Minneapolis, MN USA; 6 Department of Biomedical Engineering, University of Minnesota, Minneapolis, MN USA

**Keywords:** Transdermal osseointegrated implant, Mesenchymal stromal cells, Integumentary tissues, Cell adhesion, Titanium surfaces

## Abstract

**Background:**

Transdermal osseointegrated prosthesis have relatively high infection rates leading to implant revision or failure. A principle cause for this complication is the absence of a durable impervious biomechanical seal at the interface of the hard structure (implant) and adjacent soft tissues. This study explores the possibility of recapitulating an analogous cellular musculoskeletal-connective tissue interface, which is present at naturally occurring integumentary tissues where a hard structure exits the skin, such as the nail bed, hoof, and tooth.

**Methods:**

Porcine mesenchymal stromal cells (pMSCs) were derived from nine different porcine integumentary and connective tissues: hoof-associated superficial flexor tendon, molar-associated periodontal ligament, Achilles tendon, adipose tissue and skin dermis from the hind limb and abdominal regions, bone marrow and muscle. For all nine pMSCs, the phenotype, multi-lineage differentiation potential and their adhesiveness to clinical grade titanium was characterized. Transcriptomic analysis of 11 common genes encoding cytoskeletal proteins *VIM* (*Vimentin*), cell–cell and cell–matrix adhesion genes (*Vinculin, Integrin β1, Integrin β2, CD9, CD151*), and for ECM genes (*Collagen-1a1, Collagen-4a1, Fibronectin, Laminin-α5, Contactin-3*) in early passaged cells was performed using qRT-PCR.

**Results:**

All tissue-derived pMSCs were characterized as mesenchymal origin by adherence to plastic, expression of cell surface markers including *CD29*, *CD44*, *CD90*, and CD105, and lack of hematopoietic (*CD11b*) and endothelial (*CD31*) markers. All pMSCs differentiated into osteoblasts, adipocytes and chondrocytes, albeit at varying degrees, under specific culture conditions. Among the eleven adhesion genes evaluated, the cytoskeletal intermediate filament vimentin was found highly expressed in pMSC isolated from all tissues, followed by genes for the extracellular matrix proteins *Fibronectin* and *Collagen-1a1*. Expression of *Vimentin* was the highest in Achilles tendon, while *Fibronectin* and *Col1agen-1a1* were highest in molar and hoof-associated superficial flexor tendon bone marrow, respectively. Achilles tendon ranked the highest in both multilineage differentiation and adhesion assessments to titanium metal.

**Conclusions:**

These findings support further preclinical research of these tissue specific-derived MSCs in vivo in a transdermal osseointegration implant model.

**Supplementary Information:**

The online version contains supplementary material available at 10.1186/s13287-021-02666-2.

## Introduction

Osseointegration is the direct apposition of bone onto a metallic implant, ideally, without fibrous tissue interposition [1]. Transdermal systems have been recently popularized for the use in patients with limb amputations. In amputees, bone anchored devices traverse the skin-bone-implant interface in order to form a direct mechanical connection between the bone and the external prosthesis [2]. Indeed, transdermal systems have revolutionized the quality of life for patients living with major limb amputations, particularly for military service members and veterans. In a national survey of wounded service members and veterans with unilateral upper limb amputations, using traditional (non-osseointegrated) socket prosthesis, it was found that nearly a third completely abandoned their prosthetic device due to skin irritation, discomfort, prosthesis weight and associated morbidities [3]. Unlike socket-based prosthesis, osseointegration results in direct load transfer to the skeleton, elimination of the need for prosthetic revisions due to residual limb shape changes, optimum control of the prosthetic movement, and minimal risk of nerve compression or skin irritation. Anchoring the prosthetic directly to bone also affords the same restitution of sensory and tactile function, osseoperception, as well as a sense of proprioception, a sensory function thought to be impossible to achieve with conventional socket prostheses [4]. All these advantages culminate in overall improved quality of life, increased functional independence, and prompt return to an active lifestyle [5, 6].

While the transdermal nature of osseointegrated implants enables the attachment of a variety of prosthetic devices, the skin-implant interface, known as the aperture, is a unique and ill-defined microenvironment that is integral to prolonged implant stability and homeostasis. The possibility of deep and superficial infection complications remains a major clinical concern for both patients and surgeons, posing a substantial barrier to widespread use. Further, excessive soft tissue motion at the aperture result in progressive exposure of adjacent soft tissues thereby increasing chances of infection, osteomyelitis, implant loosening, surgical revisions and implant failure [7].

Techniques to augment adhesion and durability of the skin and underlying connective tissue to metal at the aperture may improve the durability and long-term viability of the skin-implant ‘seal,’ resulting in reduction of infection and implant loosening. Natural transdermal structures such as nail bed, hoof and tooth, generate an effective mechanical barrier to prevent infections and create a strong, durable hard-soft tissue interface. Recapitulating an analogous cellular transdermal musculoskeletal-connective tissue interface for osseointegrated implants may be a potentially powerful strategy to reduce infection and enhance long-term clinical outcomes [8].

Mesenchymal stromal cell (MSC) therapies have become a widely explored method for treating diseases and potentiating regeneration [9]. Integration with tissue engineering approaches have successfully incorporated MSCs into biomaterial carriers to reduce immediate clearance of transplanted MSC, prolong survival and localization of these cells, thereby enhancing the long-term paracrine effector function of MSCs [10]. However, predicting clinically meaningful outcomes has been difficult for many reasons, such as MSC heterogeneity based on tissue and isolation methods, thus necessitating robust characterization prior to delivery [11–13]. Given the multi-lineage differentiation potential of MSCs into bone, cartilage and fat tissues, our approach for recapitulating cellular transdermal barriers is to deliver MSCs in a biomaterial construct to form a flexible and durable impervious seal.

Here, as a necessary first step toward translation, we characterized the phenotype, multi-lineage differentiation potential and adhesiveness of various tissue-derived porcine MSCs (pMSCs) to clinical grade titanium. The tissues selected for this study can be categorized into two broad groups: 1) those present at the junction of a hard and soft tissue (such as periodontal ligament), present at the interface of the tooth cementum and the supporting bone, and the superficial flexor tendon (anchoring the hoof bone to the soft tissue) and 2) connective tissues such from dermis, adipose tissue and Achilles tendon tissue. We hypothesized that multi-lineage differentiation and metal adhesion properties of porcine integumentary and associated connective tissues would be different than muscle and bone marrow-derived MSCs commonly used for MSC therapy. Porcine musculoskeletal tissues are similar to humans with respect to clinical, histological and immunohistological features associated with tissue regeneration and cutaneous skin healing [14, 15]. Findings from this study will subsequently be tested in vivo using a porcine transdermal implant model developed by our team for studying wound healing and infections at percutaneous titanium (Ti) implant sites. Overall, our detailed characterization and comparative analysis of a broad panel of integumentary and connective tissue-derived MSCs will enable selection of best cell candidates and contribute to developing therapies to enhance transdermal system outcomes.

## Materials and methods

### Animals and tissue collection

Tissues were aseptically collected under institutional tissue sharing protocols from 6-month-old female Gottingen, Yucatan or Yorkshire minipigs, *Sus scrofa domesticus,* immediately after humane euthanization, per approved IACUC guidelines. Bone marrow cells were harvested and isolated from ribs or iliac crest by marrow aspiration using a heparin pre-coated syringe fitted with a Jamshidi bone marrow biopsy aspiration needle. For all other tissues the collection sites were aseptically cleansed prior to incision with ethanol and iodine wipes, then irrigated with phosphate-buffered saline (PBS; Gibco, Gaithersburg MD) supplemented with 200 U/ml penicillin, 200 µg/ml streptomycin and 250 µg/ml amphotericin B (Gibco, Gaithersburg MD). Details regarding tissue description and the sites of tissue collection are listed in Additional file 1: Table S1.

### Cell culture

Heparinized bone marrow was washed twice, treated with ACK lysing buffer to deplete RBCs, and resuspended in complete MSC stromal growth medium (cMSC-GM; DMEM-F12 (1:1); Sigma Aldrich, St. Louis, MO), supplemented with 2% FBS and antibiotics (200 units/ml penicillin, 200 µg/ml streptomycin and 250 µg/ml amphotericin B; Gibco, Gaithersburg MD). Periodontal ligament tissue was scraped off the outer surface of the molars. Harvested tissues were placed in a 10 cm tissue culture dish (Fisher Scientific, Waltham MA) containing 5–7 ml of cMSC-GM, then finely minced into a slurry of small pieces (< 1mm^3^) using fine sterile scissors. Refer to Additional file 1: Table S1 for enzyme concentrations and tissue digestion conditions. Digested tissue cell suspensions were washed, resuspended in 15 ml of cMSC-GM and filtered through 100, 70 and 40 µm cell strainers sequentially (Fisher Scientific) to obtain a single-cell suspension. Cells were placed into a 10-cm sterile tissue culture dish. Cultures were maintained at 37 °C in a humidified atmosphere containing 5% CO_2_ until 70–80% confluent.

Dermal tissue was processed differently to preserve viability [16]. Briefly, the epidermal skin layer was gently scraped using a scalpel blade to remove hair prior to overnight enzymatic digestion. Digested skin was vigorously vortexed to mechanically remove the epidermal layer. Dermal tissue explants (~ 4 cm × 2 cm) were cut into small pieces, and evenly placed on a 10-cm tissue culture dish (Fisher Scientific). A minimum volume of cMSC-GM was added and replenished every 3–5 days near the explants dropwise, ensuring no tissue movement.

### Osteogenic differentiation

For induction of osteogenic differentiation, Passage-2 pMSCs resuspended in cMSC-GM were seeded in either in a 96-well plate (100 cells/well) for assessing early osteogenic differentiation via a pNPP based alkaline phosphatase assay, in a 6-well plate (1 × 10^5^ cells/well) for osteogenic gene expression studies or at 2.5 × 10^5^ cells /well in a 6-well plate for assessing long term osteogenic differentiation potential, as previously described [17–19]. Media was changed from growth media to StemPro® Osteogenic Differentiation media (‘OM’; Life Technologies, Carlsbad CA) for half of the wells 24 h post seeding, with the other half (control wells) maintained in growth media (GM). Both OM and GM were replaced every 3 days. For pNPP-based assay, media was aspirated after 7 days, washed with PBS, followed by cell lysis in 1% TritonX-100 and addition of the pNPP substrate (Sigma Aldrich). Absorbance was measured immediately at 405 nm on a kinetic mode for 20 cycles on a Tecan Infinite 200 PRO fluorometer (Tecan; Morrisville NC). Cultured pMSCs were harvested in QIAzol Lysis Reagent (Qiagen, Germantown, MD) for gene expression studies. For long term osteogenic differentiation studies, adherent cell cultures after 14–28 days of culture were fixed in 0.5% glutaraldehyde for 20 min, followed by multiple PBS washes, and mineralization (presence of calcium-rich hydroxyapatite of the extracellular matrix) was assessed by staining with 2% alizarin red solution (prepared in distilled water; pH = 4.2) for 3–5 h at room temperature. Post staining, adherent cells were washed multiple times with tap water, followed by deionized water, and imaged microscopically (Axio Observer Z1, Zeiss). For quantification, alizarin red stained calcium crystals were solubilized using 10% formic acid, incubated for 45–60 min with shaking at room temperature, and absorbance measured at 414 nm (Tecan Infinite 200 PRO fluorometer; Tecan, Morrisville NC). Osteogenic differentiation experiments were carried out for all nine tissues in triplicates; i.e., isolated from 3 different animals.

### Chondrogenic differentiation

Chondrogenic differentiation of Passage-3 pMSCs was performed using pellet cultures. Briefly, cells were expanded in GM until attaining 80% confluency, after which cells were dissociated with trypsin and re-suspended in chondrogenic differentiation medium in V-well polypropylene plates at a density of 5.0 × 10^5^ cells /well. Cells were centrifuged at 200 g for 4 min and maintained in the media for 21 days with media changes every three days. The standard chondrogenic differentiation media consisted of DMEM (high glucose) media supplemented with 1% ITS + (Sigma, USA; St. Louis, MO), 100 nM dexamethasone (Sigma, USA; St. Louis, MO), 1.25 mg/ml bovine serum albumin (Sigma, USA; St. Louis, MO), 10 ng/ml TGF-β1 (R&D systems, USA), and 0.1 mM ascorbic acid 2 phosphate (Sigma, USA; St. Louis, MO). Pellets (in triplicates) were collected either at 7 days for gene expression analysis (in QIAzol Lysis Reagent), or after 21 days for histological and quantitative assessments of differentiation. Synthesis of glycosaminoglycans (GAG) was measured to quantify chondrogenic differentiation. For GAG analysis, collected pellets (in triplicates) were digested at 56 °C in 300 μL of 1 mg/mL proteinase-K solution to digest the matrix, followed by addition of dimethyl-methylene blue (DMMB) dye. Total GAG content was determined by measuring the absorption of the molecular complex at 492 nm (Synergy TM 2, Biotek multi-mode microplate reader). Chondroitin sulphate B was used to prepare a standard curve. GAG content was normalized against the total DNA content, measured using the cell lysate following the manufacturer’s protocol (CyQuant cell proliferation assay). Briefly, diluted cell lysate was mixed with 2X GR-dye and incubated for 1 h at room temperature following which the fluorescence was measured at *λ*_ex_ of 480 nm and *λ*_em_ of 520 nm (Synergy TM 2, Biotek multi-mode microplate reader). Chondrogenic differentiation was quantified as the ratio of GAG to the DNA content (GAG/DNA). For histological analysis, collected pellets (in triplicates) were fixed in 4% paraformaldehyde for 30 min at room temperature, cryo-sectioned (30 µm thick sections) and stained with Alcian blue to assess levels of sulphated glycosaminoglycans and counterstained with Nuclear Fast Red. The set of pellets collected at day 7 post chondrogenic differentiation was used to assess the expression of three chondrogenic genes: *SOX9, Collagen-2a1* and *Aggrecan*.

### Adipogenic differentiation

Adipogenic differentiation of Passage-2 pMSCs resuspended in cMSC-GM seeded in either in a 6-well plate (1 × 10^5^ cells/well) for adipogenic gene expression studies, or at 2.5 × 10^4^ cells/well in a 6-well plate for assessing long term adipogenic differentiation potential. Media was changed from GM to AdipoQual adipogenic media (‘AM’; Obatala Sciences; New Orleans, LA) for half of the wells when cells reached 40–60% confluency, with the other half (control wells) continued in GM. Both AM and GM were replaced every 3 days. For gene expression studies, cells were harvested after 7 days in differentiation or growth media, via cell scraping, and stored at − 80 °C in 1 ml of QIAzol Lysis Reagent until further processing. For long term adipogenic differentiation studies, cultured pMSCs were harvested between 14–28 days; fixed in 0.5% glutaraldehyde for 20 min, followed by multiple PBS washes, and stained with Oil Red O Staining Solution (Obatala Sciences; New Orleans LA) for 30 min to an hour. Post staining, wells were washed multiple times with DI water and imaged microscopically (Axio Observer Z1, Zeiss). For quantification, Oil Red O-stained lipid droplets were solubilized using 100% isopropanol, incubated for 10 min with shaking at room temperature, and fluorescence measured at *λ*_ex_ of 500 and *λ*_em_ of 595 nm (Tecan Infinite 200 PRO fluorometer; Tecan, Morrisville NC). All adipogenic differentiation experiments were carried out for nine tissues in triplicates; i.e. each of the nine tissues isolated from 3 different animals.

### Gene transcripts associated with cell adhesion

Adhesion gene profiling was conducted using Passage-5 pMSCs (1 × 10^5^ cells/well) grown in cMSC-GM, OM, CM and AM for 7-days. Adherent cell cultures were washed twice with PBS, scraped from the plate, and pelleted by centrifugation, stored at − 80 °C in 1 ml of QIAzol Lysis Reagent (Qiagen, Germantown MD). Total RNA was isolated using the miRNAeasy Mini Kits (Qiagen). Quantitative and qualitative evaluation measurements of RNA samples were conducted using NanoDrop and Agilent 2100 Bioanalyzer instruments (Thermo Scientific, Waltham, MA). cDNA was synthesized using a high-capacity cDNA synthesis kit (Applied Biosystems, Foster City, CA) and qPCR was carried out using a Syber Green based detection (BioRad, Hercules, CA) on the Quantstudio (QuantStudio 7 Flex Real-Time System; Applied Biosystems, Waltham, MA). The list of primers and sequences used for this study have been listed in Additional file 1: Table S2 with β-actin as a housekeeping gene.

### Immunophenotype

Passages 3–5 pMSCs were harvested for flow cytometric analysis. pMSCs were washed once with room temperature PBS, incubated with 15 mM EDTA in PBS solution for 12 min at 37 °C and detached off the plate. Detached cells were washed in DMEM media supplemented with 10% FBS, centrifuged and counted. Aliquots of 2 × 10^5^ cells were suspended in 2 ml of Flow Cytometry Staining buffer (eBioscience™, Invitrogen, Waltham, MA), washed (centrifugation for 5 min at 1400 rpm). Cell pellets were resuspended in 100 µl of FACS buffer then incubated on ice for 30 min with FITC-conjugated anti-CD44 (#MA1-10228; Invitrogen, Waltham, MA), anti-CD90 (#A15761; Invitrogen), and anti-CD29 (#MA1-19566; Invitrogen, Waltham, MA), and anti-CD105 (#NB11081749; Novus Biologicals, Centennial, CO). FITC-conjugated isotype matched IgG1 (#50-204-9474; Cell Signaling Technology, Danvers, MA) and IgG2 (#PA5-33239, Invitrogen) were used as controls. After 30 min of incubation in the dark, cells were washed twice with 2 ml of Flow Cytometry Staining buffer and then resuspended 300 µl of cold FACS buffer. To detect dead cells, 1ul of SYTOX™ Blue Dead Cell Stain (Invitrogen, #S34857) was added to the cells 5 min prior to being analyzed using a BD LSRII Flow Cytometry System (BD BioSciences, Rockville, MD). Viable cells were gated in a dot plot of forward versus side scatter signals wherein 10,000 gated events were acquired over a log fluorescence scale. Flow cytometric data were analyzed, and histograms generated using FlowJo software version 10 (TreeStar Inc, Ashland, OR).

### Cell adhesion experiments on titanium alloy

#### Intracellular focal adhesion quantification

Passages 2–3 cells were seeded on Ti-6Al-4 V (Ti) polished to a 20–60 nm colloidal silica finish (President Titanium, Hanson, MA) and glass disks (Harvard Apparatus, Holliston, MA) at 1 × 10^3^ cells/disk for 4 h, 1 day, and 3 days. NIH-3T3 (CRL-1658, ATCC, Manassas, VA) embryonic murine fibroblasts (hereafter, NIH-3T3 fibroblasts) which adhere, proliferate and migrate on metal and glass surfaces served as controls Overmann [20]. Cell spreading and focal adhesion characteristics were determined with immunofluorescence. Seeded disks (*n* = 10) were washed in PBS (Gibco), fixed in 4% paraformaldehyde for 10 min, permeabilized with 0.1% Triton X-100 in PBS, blocked in 5% bovine serum albumin (BSA), followed by overnight anti-vinculin primary antibody staining (MAB3574; MilliPore Sigma, Burlington, MA), incubation with secondary antibody (ab97037; Abcam, Cambridge, UK) in PBS for 1 h at room temperature, followed by incubation with 4’,6-diamidino-2-phenylindole (DAPI; 300 nM in PBS; D9542; Fisher Scientific, Waltham, MA) for 10 min. After each step, the cells were rinsed in PBS. Disks were mounted with ProLong (Fisher) and imaged (Olympus FV1000, Tokyo Japan or Leica DM 6B, Wetzlar Germany). Cytoskeletal architecture was assessed by rhodamine-phalloidin (R415, ThermoFisher Scientific, Waltham, MA) based actin staining on the remaining disks (*n* = 5). These slides were processed using the same protocol as described above, counterstained with DAPI and mounted. ImageJ (NIH) was used for image analysis of all fluorescent images, with 5 fields of views (FOVs) per sample. Proliferation (number of DAPI-positive nuclei), cell surface area per FOV, individual cell area, vinculin (focal adhesion) intensity per FOV, average vinculin (focal adhesion) intensity per cell, and average vinculin (focal adhesion) intensity per cell area were calculated and normalized to NIH-3T3 fibroblasts on Ti at 4 h for each measure to account for differences in microscope usage. All experiments were repeated on pMSCs derived from at least 3 animals.

#### Cell proliferation and viability

A colorimetric assay (CCK-8) was used to compare the proliferation and viability of pMSCs on both Ti and control glass. At 4 h, 1 day and 3 days, seeded disks (*n* = 7; both Ti and glass) were washed thrice in PBS to remove weakly adherent cells, transferred to a virgin 48 well-plate, and incubated for 2.5 h in a 1:10 dilution of CCK-8 solution (Dojindo Laboratories, Japan) in culture media. Afterward, 100 μL of the CCK-8/media solution was transferred to a 96-well plate for absorbance reading (Synergy, BioTek—450 nm) expressed as optical density (O.D.). A blank, comprising of CCK-8/media solution, without cells was used to subtract background absorbance. All experiments were repeated on pMSCs derived from at least 3 animals.

#### Centrifugal assessment of cell adhesion

A centrifuge-based assay was used to apply shear to pMSCs to quantify their physical adhesion to titanium [21]. pMSCs (5 × 10^3^) were seeded onto polished titanium disk surfaces in 48-well plates for 48 h then gently washed thrice with PBS to remove non-adherent cells. Oral keratinocytes (OKF6/TERT-2; BWH Cell Culture and Microscopy Core, Boston, MA) were used as a positive control given their strong adhesion to titanium [22]. One set of disks (*n* = 3) were immediately removed and fixed in 4% paraformaldehyde (hereafter, control-pre). The second set (*n* = 3) were inserted into a three-dimensionally (3D) printed (Dental Resin SG, Formlabs, Somerville, MA) disk-holder (Additional file 1: Fig. S1). This holder fits inside a 48-well such that disks (one disk per holder per well) are oriented perpendicular to the ground when centrifuged. Wells were filled with culture media and then centrifuged (3 min) at 350 g or 500 g (*n* = 3 per sample). Control disks (*n* = 3 sample; hereafter, control-post) were treated identically, but placed next to the centrifuge (5810R; Eppendorf, Hamburg Germany) while the other two groups were centrifuged. Disks were then subsequently stained with DAPI for 15 min at room temperature and imaged (Lecia DM6 B). ImageJ (NIH) was used for quantification of nuclei in three random field of views per disk.

### Statistical analysis

GraphPad Prism (v8; GraphPad Software, San Diego, CA) was used for statistical analyses and graphic drawings. Experimental data were evaluated by one-way ANOVA, followed by the Tukey HSD multiple comparison test. A *t*-test with False Discovery Rate (FDR) correction (*Q* = 1%) was used to compare titanium vs. glass for each measure. An ANOVA with Dunnett’s multiple comparison was used to compare the various pMSCs cell types to NIH-3T3 fibroblasts (control group) for each measure. Inter-cell population differences between control-post, 350 g, and 500 g were detected with a two-way analysis of variance with a Dunnett correction where the control mean (null) was set as keratinocytes. A value of *p* < 0.05 was considered as significant difference.

## Results

### Isolation and culture of porcine mesenchymal stromal cells (pMSCs) derived from integumentary and connective tissues

**Fig. 1:**
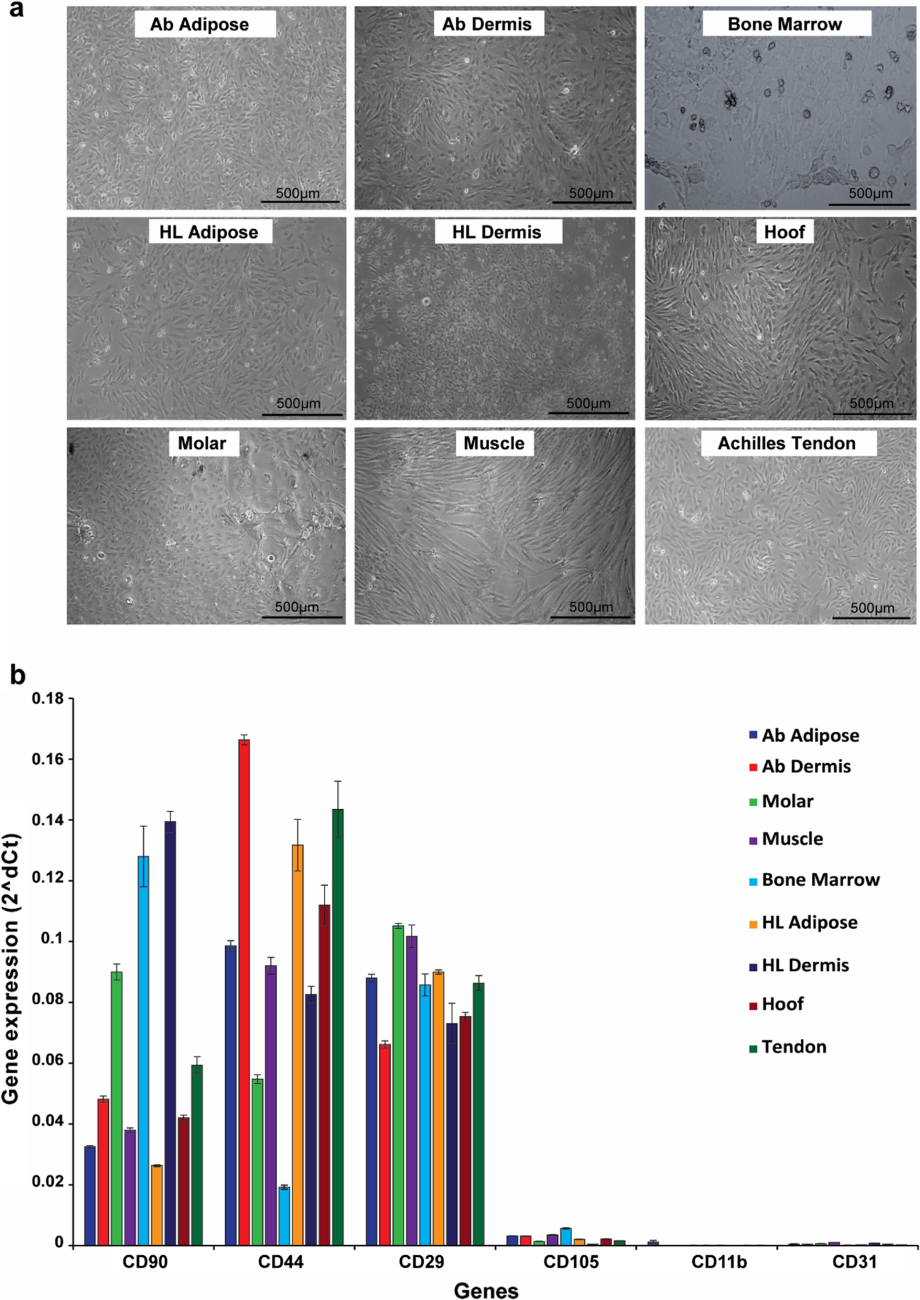
Isolation and phenotypic characterization of nine porcine tissue-derived mesenchymal stromal cells (pMSCs). **a** Representative phase contrast images (50 × magnification) of Passage-0 cells, 10 days after isolation from the different porcine integumentary and connective tissues as specified. For all figures, *Ab* Abdominal, *HL* Hind limb, *Hoof* Hoof-associated superficial flexor tendon, *Molar* Molar-associated periodontal ligament, *Muscle* Gastrocnemius muscle **b** Phenotypic characterization of the pMSCs by gene transcripts of MSC (*CD90, CD44, CD29, CD105*), hematopoietic (*CD11b*) and endothelial (*CD31*) markers in Passage-2 cells cultured in growth media. β-Actin was used for normalization of gene expression. Data presented as mean ± SEM (*n* = 3; *p < 0.05)

As shown in Fig. 1a, a population of “fibroblast-like” cells were successfully isolated from nine distinct porcine tissues, comprising either integumentary (periodontal ligament, hoof-associated flexor tendon) or connective (Achilles tendon, dermis, adipose) tissues, in addition to bone marrow and muscle, using enzymatic and mechanical dissociation as detailed in Methods and Additional file 1: Table S1. Except for dermal tissue, single cell suspensions obtained from post-tissue digestion were seeded on tissue-culture treated polystyrene (TCPS). Dermis-resident cells were derived from epithelial layer-depleted skin explants, seeded on TCPS. By day five, distinct foci (clusters of 5–10 cells) of plastic adherent cells were evident in all single-cell suspension derived cultures and retained high viability and proliferation potential after extended in vitro expansion. Outgrowth of pMSCs was greatest in adipose tissue and least from dermal tissue; however, by Passage-2 growth across all cell type were comparable through Passage-5. In contrast, pMSCs derived from periodontal ligament exhibited a mixed population of fibroblast like and spherical cells, while pMSCs derived from hind limb dermis often contained a mixture of fibroblast and cobblestone shaped cells. pMSCs isolated and expanded from all three pig strains (Gottingen, Yucatan and Yorkshire) revealed similar characteristics.

### Gene expression of pMSC-surface markers

We used RT‐PCR to profile the mRNA expression patterns of MSC-specific cell surface markers in Passage-2 cells (Fig. 1b). All the nine tissue-derived cells expressed high levels of mRNA transcripts for porcine cell surface markers whose expression can be used to functionally characterize isolated pMSCs, such as *CD29*, *CD44* and *CD90*, while negative for hematopoietic (*CD11b*) and endothelial (*CD31*) gene transcripts [23]. The expression of the *CD105* transcript was not strongly detected in any of the nine pMSCs comparing to others MSC markers studied but still significantly higher than the expression of hematopoietic and endothelial markers (Fig. 1b). The expression of *CD105* as a pMSC marker in pigs is still controversial [24, 25]. The pattern of gene transcript expression for the pMSC-specific markers varied across the nine tissues; molar-derived periodontal ligament (Molar), bone marrow and hind limb (HL) dermis-derived cells expressed significantly higher levels of the *CD90* transcript, compared to the other cell types; while abdominal (Ab) dermis, hind limb adipose and tendon-derived cells expressed significantly high levels of the *CD44* transcript. Most of the cell types expressed comparable levels of *CD29* gene transcript expression, although the expression levels in molar and muscle-derived cells were significantly higher than in all other cell types.

### Cell surface immunophenotype of pMSCs

**Fig. 2:**
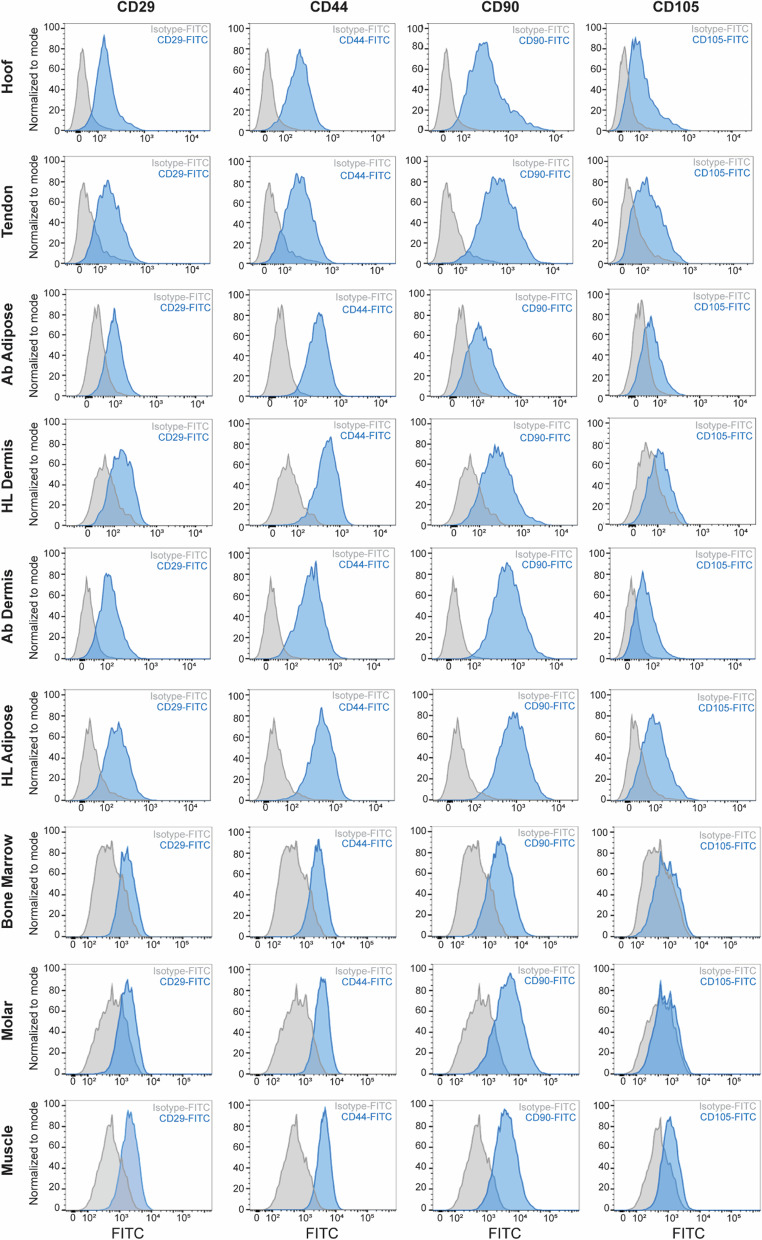
Cell surface marker immunophenotype of porcine tissue-derived mesenchymal stromal cell (pMSCs) using flow cytometric analysis. Flow cytometric analysis of cell surface protein expression on pMSCs using FITC-conjugated anti CD29, CD44, CD90, and CD105 antibodies. Cell surface log fluorescent measurements were obtained on 10,000 viable cells using forward versus side scatter (FSC vs SSC) gating. Representative fluorescent histograms of cells stained with isotype matched (control) and MSC surface marker antibodies are shown

In order to confirm the gene expression data, cell surface protein expression was assessed using flow cytometric analysis. In comparison to median fluorescent signals on isotype control stained pMSCs, we observed that all of the pMSC-derived cell types strongly expressed CD29 (3–6 fold increase), CD44 (5–14 fold increase) and CD90 (4–26 fold increase), albeit the expression of CD105 (1.5–4 fold increase) was significantly less (Fig. 2). These data and the mRNA transcript results demonstrate that pMSCs isolated from different pig tissues share a similar mesenchymal cell surface marker phenotype (CD29^low^, CD44^high^, CD90^high^ and CD105^dim^) immunophenotype.

### pMSCs exhibit trilineage differentiation potency

Further functional characterization of the pMSCs was carried out by assessing the multilineage potential of these cells during expansion. On induction of differentiation conditions, all pMSCs lines differentiated into osteoblasts, adipocytes and chondrocytes, albeit at varying degrees, under specific culture conditions.

#### Osteogenic differentiation

**Fig. 3:**
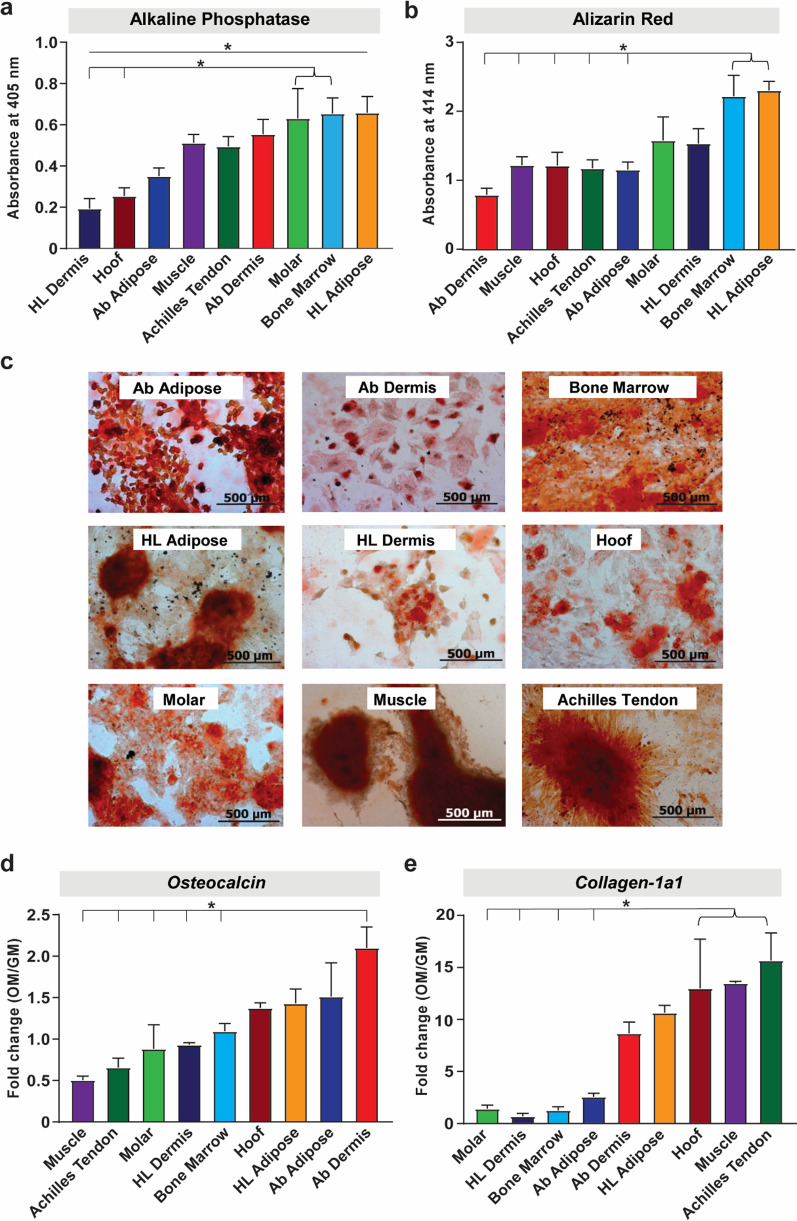
Assessment of in vitro osteogenic differentiation of pMSCs. **a** Quantification of early osteogenic differentiation potential of pMSCs cultured in osteogenic induction media for 7 days, assessed by pNPP-based alkaline phosphatase activity assay. **b** Quantification of alizarin red stain-based assessment of late osteogenic differentiation of cells cultured in osteogenic induction media for 14–28 days. **c** Representative phase contrast images (50 × magnification) of cells stained with alizarin Red. **d**–**e** Fold change in gene expression of osteogenic transcription factors, *Osteocalcin (BGLAP)* (**d**) and *Collagen-1a1* (**e**) in cells cultured in osteogenic media (OM) normalized to their expression under growth media (GM) for 7 days. β-Actin was used for normalization of gene expression. Data presented as mean ± SEM (*n* = 3; *p < 0.05)

pMSCs derived from the adipose tissue of the hind limb (HL) adipose, bone marrow and molar-associated periodontal ligament demonstrated the highest potential of early osteogenic lineage commitment, as assessed by the alkaline phosphatase activity assay (Fig. 3a). In long term culture under extended osteogenic differentiation conditions, calcium deposition as a late indicator of osteogenic differentiation, was markedly increased in HL adipose and bone marrow-derived pMSCs (Fig. 3b–c). Analysis of osteoblast-specific genes in these cells after one week in osteogenic differentiation media demonstrated relatively high expression level of *BGLAP* (*Osteocalcin)* and *COL1A1 (Collagen-1a1)* genes in HL adipose-derived pMSCs (Fig. 3d, e), when compared to cells cultured in growth media. The expression level of these genes in HL adipose-derived pMSCs was comparable to expression in pMSCs derived from other tissues, such as Achilles tendon, muscle, hoof-associated tendon, abdominal adipose and dermal tissues; indicating potent osteoblastic differentiation potential of all these cell types. In addition, the same osteogenic induction signals resulted in induction of other pro-osteogenic genes in the different tissue-derived pMSCs. For example, there was pre-dominance of expression of *BGLAP* (Fig. 3d) and *RUNX2 (Runt-related transcription factor 2*) (Additional file 1: Fig. S2a) in abdominal dermis and abdominal adipose; *COL1A1* (Fig. 3e) and *BSP (Bone sialoprotein)* (Additional file 1: Fig. S2b) in muscle, tendon and hoof-associated superficial flexor tendon pMSCs, and *OPN* (*Osteopontin)* in bone marrow and molar-derived pMSCs (Additional file 1: Fig. S2c).

#### Chondrogenic differentiation

**Fig. 4:**
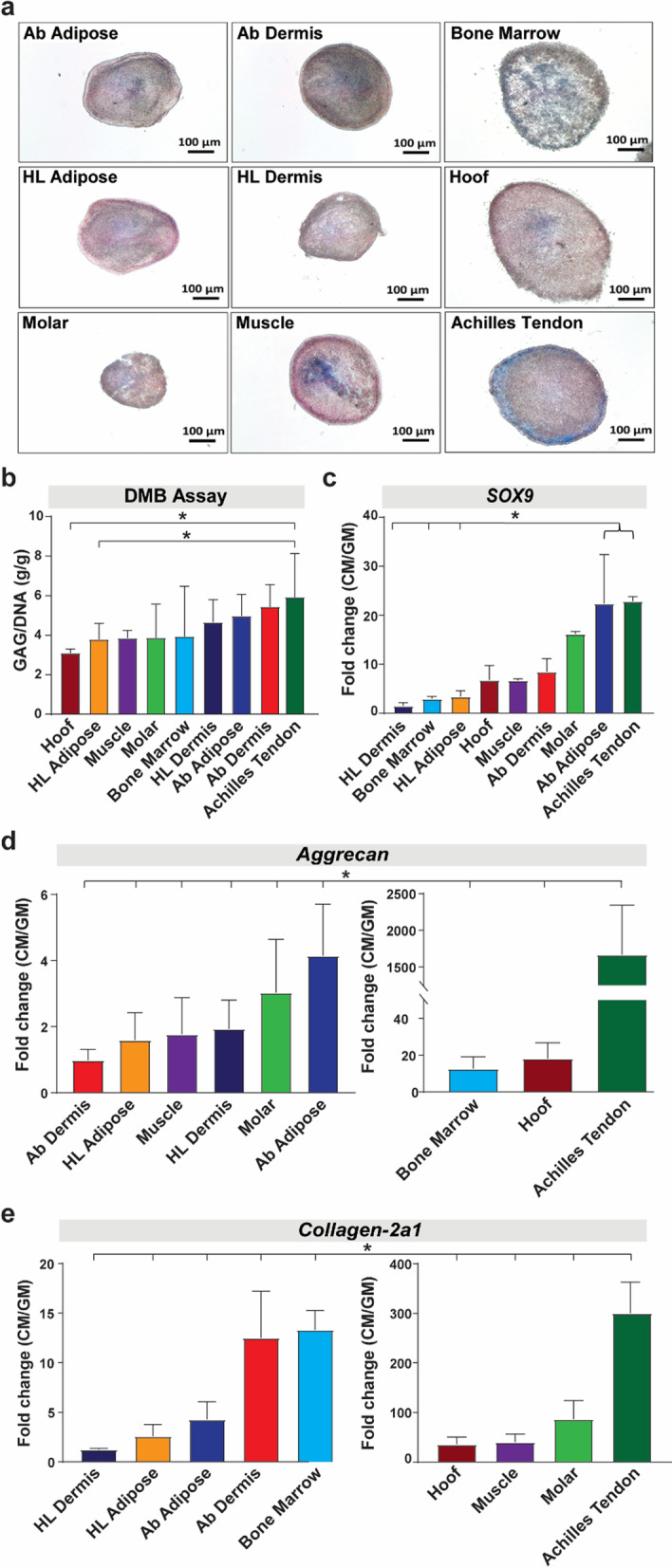
Assessment of in vitro chondrogenic differentiation of pMSCs. **a** Histological staining (Alcian blue/Nuclear Fast Red) of pellets derived from pMSCs of different tissue sources cultured in chondrogenic media after 21 days in culture (50 × magnification). **b** Quantification of chondrogenic differentiation by calculating the levels of glycosaminoglycans (GAG) secreted by cells under chondrogenic differentiation conditions, normalized to the cell density in the pellets (DNA) after 21 days in culture. Data presented as mean ± SD (*n* = 3 biological replicate with three pellets per replicate). *p < 0.05 and **p < 0.01 **c–e** Fold change in gene expression profiling of chondrogenic genes, *SOX9* (**c**) *Aggrecan* (**d**) and *collagen II* (**E**) in pMSCs cultured in chondrogenic media (CM) vs growth media (GM) for 7 days. β-Actin was used for normalization of gene expression. Data presented as mean ± SEM (*n* = 3; *p < 0.05)

As shown in Fig. 4, Achilles tendon-derived pMSCs had the greatest chondrogenic differentiation capacity, as assessed by Alcian blue staining (Fig. 4a) and measurement of normalized glycosaminoglycans (GAG) (Fig. 4b). This was confirmed by gene expression analysis of chondrocyte-specific genes, such as *ACAN* (*Aggrecan), COL2a1 (Collagen-2a1) and SOX9 (SRY-box transcription factor 9)* in pMSCs harvested after 7 days in pellet cultures (Fig. 4c–e). Achilles tendon-derived pMSCs demonstrated the highest expression of these pro-chondrogenic genes compared to the corresponding cells cultured in growth media.

#### Adipogenic differentiation

**Fig. 5:**
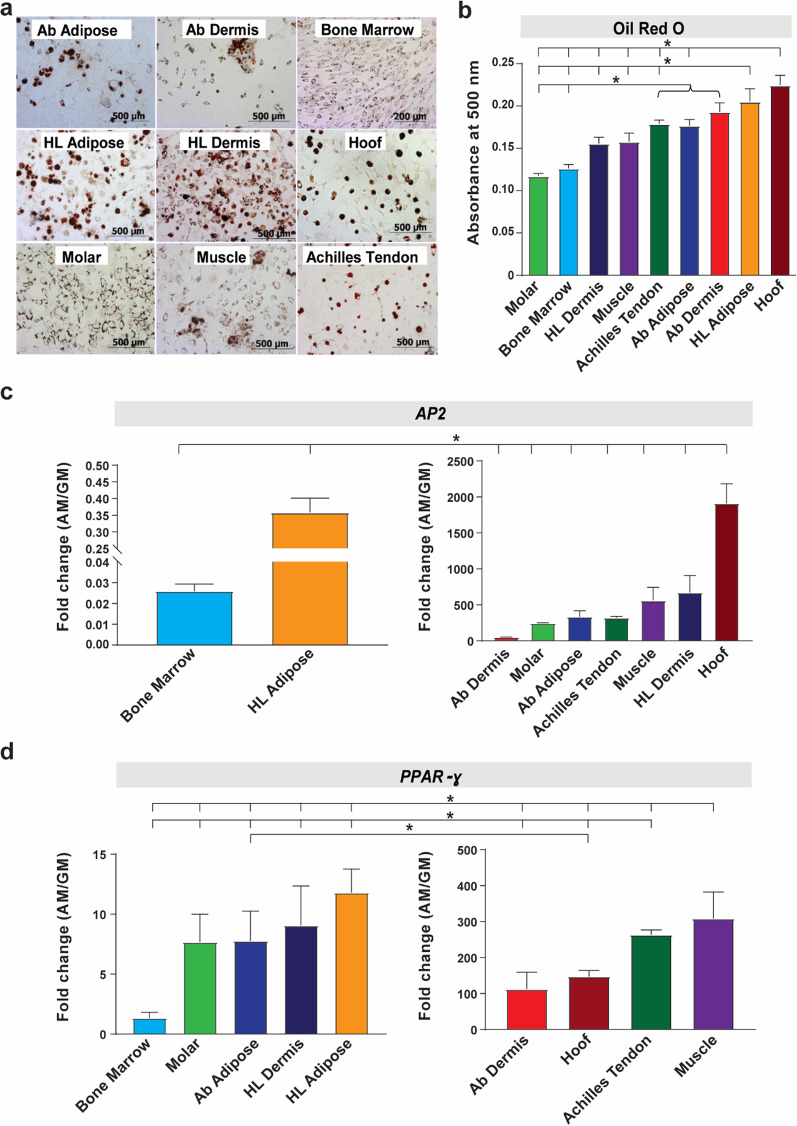
Assessment of in vitro adipogenic differentiation of pMSCs. **a** Histological staining (Oil Red O) of the nine pMSCs cultured in adipogenic media after 21–28 days in culture (50 × magnification). **b** Quantification of intracellular lipid droplet formation by measuring absorbance of solubilized Oil Red O at 500 nm. **c**–**d** Fold change in gene expression of the adipogenic transporter *AP2* (**c**) and receptor *PPAR-ɣ* (**d**) in pMSCs cultured in adipogenic media (AM) versus growth media (GM). β-Actin was used for normalization of gene expression. Data presented as mean ± SEM (*n* = 3; *p < 0.05)

All the tissue-derived pMSCs populations demonstrated varied low-to-modest levels of intracellular lipid droplets accumulation (Fig. 5a). Isopropanol-based solubilization of the lipid droplets, followed by spectrophotometric quantification, indicated that hoof-associated superficial flexor tendon-derived pMSCs had the highest level of adipogenic differentiation, followed by HL adipose tissue-derived pMSCs (Fig. 5b). This was corroborated by the highest upregulation of the adipogenic transporter, *AP2 (Adipocyte protein 2),* in hoof-associated superficial flexor tendon pMSCs observed after a week in adipogenic induction medium (Fig. 5c). While hoof-associated superficial flexor tendon pMSCs also had high expression of another well-known adipogenic transcription factor, *PPARɣ (Peroxisome proliferator activated receptor gamma)* (Fig. 5d), muscle and tendon-derived pMSCs also demonstrated significantly high upregulation of *PPARɣ* in early stages of adipogenic induction.

### Profiling of cell adhesion-related genes in pMSCs

**Fig. 6:**
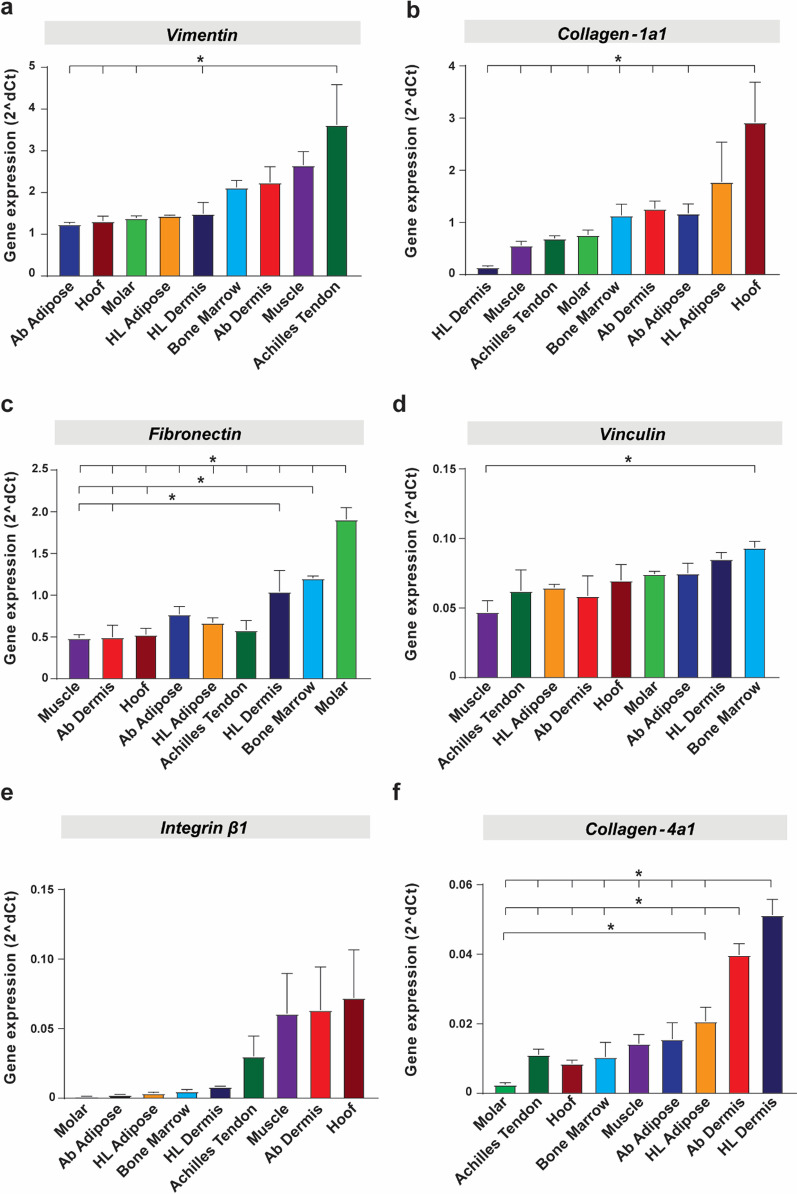
Expression profiling for a panel of adhesion genes in nine pMSCs. Syber Green based qRT-PCR for a panel of 6 adhesion genes in Passage 5 pMSCs cultured in growth media. **a** *Vimentin* **b** *Col1agen-1a1* **c** *Fibronectin* **d** *Vinculin* **e** *Integrin β1*, and **f** *Collagen-4a1*. β-Actin was used for normalization of gene expression. Data presented as mean ± SEM (*n* = 3; *p < 0.05)

Others have shown that surface modifications (porosity, surface roughness, scaffold coatings) of the titanium (Ti) metal can improve cell adhesion and osseointegration [20]. One of the critical parameters for selection of a promising cell type to reinforce the aperture is its adhesion strength to metal, scaffold and surrounding tissue. Such adhesion may also result in MSC tissue retention. Thus, non-stimulated pMSCs were profiled for inherent differences in expression of structural and adhesion-related genes. Using qRT-PCR based expression, an assessment of the expression of 11 common genes encoding cytoskeletal proteins *VIM* (*Vimentin*), cell–cell and cell–matrix adhesion genes (*Vinculin, Integrin β1, Integrin β2, CD9, CD151*), and for ECM genes (*Collagen-1a1, Collagen-4a1, Fibronectin, Laminin-α5, Contactin-3*) in early (Passage 2) and late (Passage 5) cells was performed. At Passage 5, all pMSCs showed high levels of expression of *Vimentin,* followed by *Collagen-1a1, Fibronectin, Vinculin, Integrin β1*, *Collagen-4a1* (Fig. 6) and *CD9, CD15, Laminin-α5, Contactin-3* and *Integrin β2* (Additional file 1: Fig. S3). Achilles tendon-derived pMSCs had the highest expression level of vimentin. Among all the cell types, hoof-associated superficial flexor tendon pMSCs expressed high levels of the majority of adhesion-related genes tested (6/11; *Collagen-1a1, Integrin β1, CD9, CD151, Laminin-α5, Contactin-3),* followed by muscle-derived pMSCs (5/11).

### pMSCs adhesion to titanium alloy surface

**Fig. 7:**
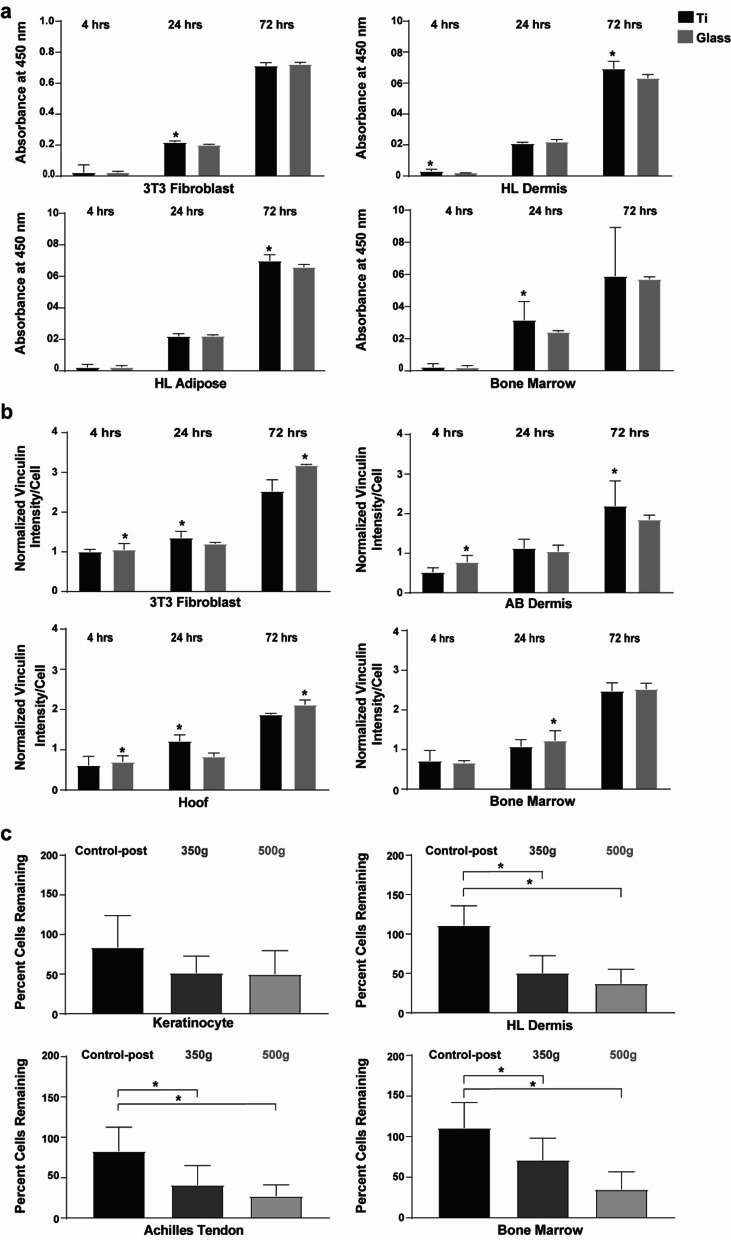
Adhesion potential of pMSCs to titanium. **a** CCK8-based assessment of pMSCs proliferation and viability demonstrating significantly higher values on titanium versus glass surface at 4, 24 and 72 h. NIH-3T3 has been used as a positive control. (Ti: Titanium; *n* = 3; *p < 0.05 between Ti and Glass) **b** Measurement of intracellular levels of the focal adhesion protein vinculin in pMSCs on titanium versus glass surface at 4, 24 and 72 h. NIH-3T3 has been used as a positive control. (Ti: Titanium; *n* = 3; *p < 0.05 between Ti and Glass) **c** Centrifugation based functional test of pMSCs adhesion to titanium, where control-post is the sham group; 350 g and 500 g are the two centrifugal forces tested. Keratinocytes have been used as a positive control. (*n* = 3; *p < 0.05)

To further characterize the potential of pMSCs strengthening the skin-implant (*i.e.*, Ti) interface, a CCK-8 based metabolic activity assay, measuring cell adhesion, proliferation and viability on titanium or glass control surfaces, was carried out. Here, we compared pMSCs to control NIH-3T3, whose proliferation on Ti is well described [26], and typical bone marrow pMSCs. These results for the top two performing pMSCs demonstrated significantly higher proliferation and viability of hind limb dermis-derived pMSCs on titanium as early as 4 h (Additional file 1: Fig. 7a), followed by hind limb adipose and bone marrow-derived cells, with significantly higher values on titanium at 72 h and 24 h with respect to glass, respectively. Molar-derived pMSCs also had high cell proliferation and viability on titanium versus glass surface at 72 h (Additional file 1: Fig. S4). As shown in Fig. 7b, abdominal dermis-derived pMSCs cultured on titanium demonstrated significantly high levels of intracellular focal adhesion marker vinculin levels when compared to glass at 72 h. Focal adhesions are known to mediate cell-Ti adhesion and proliferation [27]. This was followed by hoof-associated superficial flexor tendon pMSCs cultured on titanium, with significantly higher vinculin levels compared to cells cultured on glass at 24 h (Fig. 7b). All the other cell types generally demonstrated higher vinculin intensity on glass surface (Additional file 1: Fig. S5). Finally, a stringent functional test of the pMSCs’ adhesion to titanium was carried out by subjecting the nine pMSCs seeded on titanium disks to centrifugal forces of 350 g and 500 g, followed by assessing the percentage of cells that remained adhered to titanium post centrifugation. The positive control, keratinocytes with their well described adhesion to Ti [22], had no significant change in percentage of adhered cells on titanium pre- and post-centrifugation (Fig. 7c). However, post centrifugation titanium surfaces showed significantly fewer attached cells for all tissue-derived pMSCs (Fig. 7c and Additional file 1: Fig. S6). Bone marrow-derived pMSCs had the lowest decrease in percentage of cells adhered to titanium post centrifugation at 350 g. HL dermis and Achilles tendon-derived pMSCs had the highest remaining percent amongst the other pMSCs at 500 g (Fig. 7c).

## Summary of key functions tested

A summary table (Table 1) was created to rank the top 3 tissues for each assessment (tri-lineage differentiation, adhesion gene expression, focal adhesion to Ti, proliferation on Ti, and adherence to Ti after centrifugation). The number of times a pMSCs appeared in the list was summed to create an overall ranking, where hoof-associated superficial flexor tendon pMSCs appeared the most.

**Table 1:**
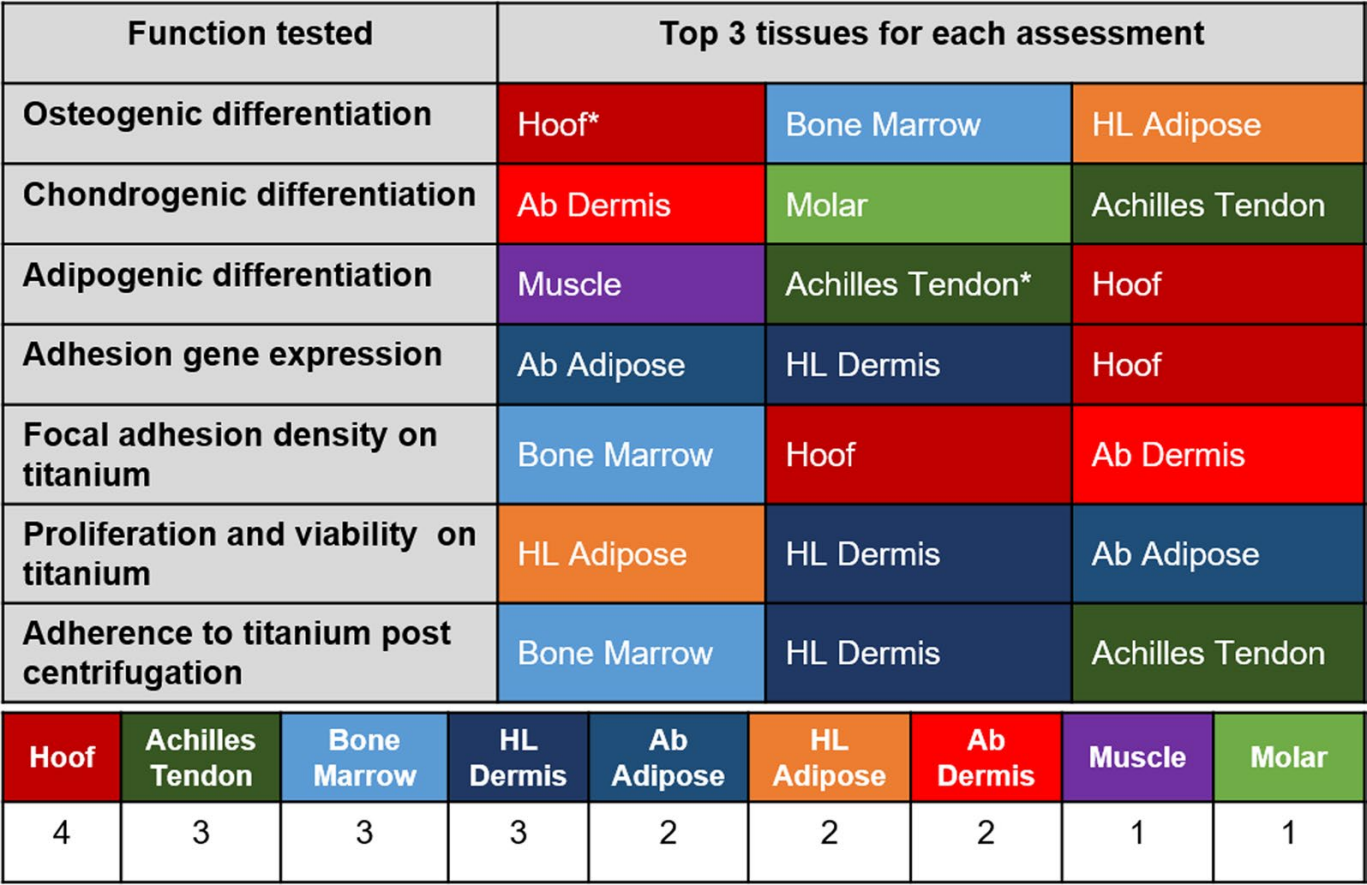
Summary and ranking of pMSCs potential for application to transdermal device therapies.

(Top) Each row represents the cellular characteristic/function studied, and the columns indicate the top 3 pMSCs (in no particular order) with highest values for each function tested. * Based on gene expression results. (Bottom) Summarized table indicating the number of times a given pMSCs appears in the top 3 list

## Discussion

This is the first comprehensive study, in our knowledge, which reports detailed characterization and comparative analysis of a broad panel of porcine integumentary and connective tissue-derived mesenchymal stromal cells. Indeed, the bulk of literature on pMSCs and their preclinical uses in the porcine model are restricted to bone marrow and adipose-derived cells [28–32], with very few reports on the isolation of multipotent pMSCs from porcine skeletal muscle [33–35], dermis [36, 37], and periodontal ligament [38]. While several reports used decellularized porcine tendon as a biological scaffold to seed human tissue-derived stromal cells [39, 40], there exist limited reports on the isolation and characterization of pMSCs derived from porcine tendon tissues. These findings indicate that these tissue-derived regenerative cells can have potential applications in bioengineering a durable skin-implant interface.

Successful isolation, ex vivo expansion, and in vitro multi-lineage differentiation of plastic adherent cells with self-renewal capacity from all nine porcine tissues demonstrated the presence functionally competent MSCs within these tissues. High expression of vimentin in all these cell types corroborated the mesenchymal origin of these cells [41]. Our results indicated that these cells can be steered to any of the three lineages studied here (osteogenic, chondrogenic and adipogenic), based on the combination of i*n vitro* induction signals the cells are exposed to. However, the varying level of expression of the different cell surface marker transcripts across the pMSCs indicated tissue-selectivity of these surface proteins or their compensatory roles in regulating pMSC function. Tissue-specificity and selectivity of the nine pMSCs was also reflected in the differentiation studies, with distinct transcription factors upregulated in the different pMSCs during the early stages of differentiation. It is important to consider species-specific differences between porcine and human MSCs that came up in this study, exemplified by the dim expression of CD105 on pMSCs, which on the other hand, is a well-recognized marker for human MSCs [23, 25]. Others have reported CD105 is [42] and is not [25] expressed by pMSCs, further supporting tissue-specificity. Here, we were able to detect low levels of mRNA gene transcript and cell surface protein expression of CD105.

Cell survival and proliferation assays demonstrated the ability of the different pMSCs to withstand forces on clinical grade titanium alloy, further supporting preclinical research of these cells in vivo. Centrifugation-based functional adhesion test of the pMSCs on titanium indicated that some cell types are more sensitive to handling than the others. For example, muscle-derived pMSCs seeded on titanium disks detached more than others at 500 g centrifugation test indicating that once seeded on titanium, these cells may not be amenable to additional manipulations and handling; thereby making them unfit for clinical purposes. Practical considerations associated with the ease of manipulation of cells are highly relevant as future steps are taken towards translation of this approach into a clinical therapy.

We ranked the nine sources of pMSC-derived with regard to osteogenic, chondrogenic and adipogenic differentiation capacity, expression of genes involved in cell adhesion, and their ability to adhere to and proliferate on titanium metal (Table 1). The results showed that hoof-associated superficial flexor tendon and Achilles tendon ranked the highest in both differentiation and adhesion assessments. Anatomical study of the porcine hoof region provides insights into the different tissue types, whose synchronous function is critical in imparting the strength, flexibility and mechanical stability of this region. Located right below the keratinized hoof is the modified dermis, corium, which generates cells that gradually keratinize to form the outer ‘horn’ of the hoof [43]. Superficial and deep flexor tendons attach the pedal bone inside the hoof to the muscles at the back of the leg, allowing for movement and flexing of the limb [44], providing a graded tensile strength along the tendon-hoof bone insertion junction, ranging from 200 MPa tensile modulus at the tendon end to 20 GPa tensile modulus at the bone end [45]. Right below the keratinized portion of the hoof, the fatty, digital cushion, composed of adipose tissue provides a shock absorbing and sensory function to the hoof [46]. pMSCs from Achilles tendon showed robust chondrogenic differentiation potential and relatively high resistance to high shear forces when cultured on titanium, while the cells from the hoof-associated flexor tendon demonstrated high adipogenic and osteogenic differentiation, expression of multiple adhesion-related genes, and high expression levels of the focal adhesion protein, vinculin, when cultured on titanium. These observations corroborated with the intrinsic function of tendons as the tissue connecting a hard tissue (bone) to a soft tissue (muscle), and thus the capability to withstand mechanical loads [47] such as those present at the aperture created by transdermal devices.

Despite the underlying similarities in hoof and Achilles tendon tissues, there is a possibility of the distinct anatomical locations (microenvironment) of the two tendons dictating their structure and function, which reflects in the function of the progenitor cells derived from these tissues. There is limited literature comparing the cellular and acellular features of these two tendon tissues, and what distinguishes them. Interestingly, there were substantial differences in the adhesion gene expression profiles between pMSCs derived from these two tendons, with cells from the hoof-associated superficial flexor tendon expressing significantly more cell–cell and cell–matrix adhesion genes than Achilles tendon derived progenitor cells.

Going forward, the validity of these in vitro findings needs to be tested in vivo before any decision can be taken on the ideal cell type or combination to stabilize the unique aperture created by transdermal bone anchored devices. Cell survival, proliferation and lineage-specific differentiation of transplanted donor MSCs in vivo is highly dependent on the microenvironment and timing of injection/persistence within the receipient tissue [48, 49]. While there are reports of MSCs mediating direct tissue repair in vivo [50–52], multiple studies have reported trophic factors such as growth factors, morphogens, chemokines, cytokines, extracellular vesicles and extracellular matrix proteins triggering activation and homing of recipient’s stem cells to the injury site, followed by tissue repair [49, 53–57]. Initial survival and adhesion of donor MSCs to the transplantation site is a critical parameter; however, the fate and contribution of the adhered MSCs to tissue repair and homeostasis would largely depend on their response to the local inflammatory milieu that exists at the site of injury, which in this context is the site of osseointegration [58]. A recent study indicated that ex vivo ‘priming’ of MSCs in an inflammatory microenvironment, before their transplantation to such an environment, significantly enhanced MSC survival and tissue reparative functions when transplanted in vivo y [59]. For our current study, it would be interesting to observe the fate of these tissue-specific pMSCs after priming them in ‘conditioned media’ of cultured tissue explants from the OI abutment site. More broadly, trophic factor expression and pMSCs delivery methods must be defined in our future work.

## Conclusions

We characterized nine pMSCs derived from different porcine integumentary and connective tissues, adipose and dermal tissues from the hind limb and abdominal regions, bone marrow and muscle. Hoof-associated superficial flexor tendon and Achilles tendon ranked the highest in both differentiation and adhesion assessments. Tissue-specific differences between MSCs may be exploited toward bioengineering a durable skin-implant interface to reduce failure of transdermal osseointegrated implants.

## Supplementary Information

**Additional file 1:** Supplementary Tables and Figures.

## Data Availability

All data generated or analyzed during this study are included in this article and its supplementary information file.

## Abbreviations

Ab: Abdominal
ACAN: Aggrecan
ACK: Ammonium-chloride-potassium
ALP: Alkaline phosphatase
AM: Adipogenic medium
AP2: Adipocyte protein 2
BGLAP: Bone gamma-carboxyglutamate protein
BM: Bone marrow
BSA: Bovine serum albumin
BSP: Bone sialoprotein
CCK8: Cell counting kit 8
CM: Chondrogenic media
cMSC-GM: Complete MSC stromal growth medium
COL1A1: Collagen Type I Alpha 1 Chain
COL2A1: Collagen Type 2 Alpha 1 Chain
DAPI: 4′,6-Diamidino-2-phenylindole
DMEM: Dulbecco’s modified eagle’s medium
DMMB: Dimethyl-methylene blue
DNA: Deoxyribonucleic acid
ECM: Extracellular matrix
EDTA: Ethylenediaminetetraacetic acid
FACS: Fluorescence-activated single cell sorting
FBS: Fetal bovine serum
FITC: Fluorescein isothiocyanate
FOV: Fields of views
GAG: Glycosaminoglycans
GPa: Gigapascal
GM: Growth medium
HL: Hind limb
HSD: Honestly significant difference
mRNA: Messenger RNA
NIH: National Institutes of Health
OI: Osteointegration implant
OM: Osteogenic media
OPN: Osteopontin
PBS: Phosphate-buffered saline
pMSCs: Porcine mesenchymal stromal cells
pNPP: P-Nitrophenyl phosphate
RBC: Red blood cell
RNA: Ribonucleic acid
RT-PCR: Reverse transcription- polymerase chain reaction
SEM: Standard error of the mean
SOX9: *SRY-box transcription factor 9*
Ti: Titanium
TCPS: Tissue-culture treated polystyrene
VIM: Vimentin
