# Supplementary material for: Culture and characterization of various porcine integumentary-connective tissue-derived mesenchymal stromal cells to facilitate tissue adhesion to percutaneous metal implants
Source: Stem Cell Res Ther. 2021 Dec 18;12:604. doi: 10.1186/s13287-021-02666-2 (PMC8684200; doi:10.1186/s13287-021-02666-2)
Supplement: Supplementary file 1 — Additional file 1: Supplementary Tables and Figures. [file 13287_2021_2666_MOESM1_ESM.docx]

**ADDITIONAL FILE 1: SUPPLEMENTAL MATERIAL**

| **Tissue** | **Site/tissue of collection** | **Enzyme cocktail** | **Duration of digestion** |
| --- | --- | --- | --- |
| **Bone marrow** | Sections of lower rib | NA; Heparinized bone marrow was obtained by obtained by iliac crest or rib aspiration | Not Applicable |
| **Muscle** | A segment of gastrocnemius muscle | Col I (3 mg/ml) + Col II (1.42 mg/ml) + Dispase (3 mg/ml) | 2 hours |
| **Abdominal (Ab) Adipose** | Lumbar region of the abdomen | Col I (1 mg/ml) | 2 hours |
| **Hind Limb (HL) Adipose** | Immediately below the skin from near the rear hock |  |  |
| **Achilles Tendon** | A segment proximal to the tendon-bone insertion junction | Col I (3 mg/ml)+Col II (3 mg/ml)+ Dispase (4 mg/ml) | 1.5 hours |
| **Hoof** | A segment of the hoof-associated superficial flexor tendon tissue was collected proximal to the hoof-tendon junction |  |  |
| **Abdominal (Ab) Dermis** | Lumbar region of the abdomen | Col I (0.25%) in DMEM-F12 (1:1) containing 10% FBS | 10-12 hours |
| **Hind limb (HL) Dermis** | Incised from above the Achilles tendon |  |  |
| **Molar-associated Periodontal ligament** | Intact mandibular molars, with the associated ligament tissue | Col I (3 mg/ml)+ Dispase II (4 mg/ml) | 1 hour |

**Table S1: Table presenting tissue collection sites and tissue processing protocol used for nine porcine tissues to derive the pMSCs.**

| **Gene** | **Forward Primer Sequence** | **Reverse Primer Sequence** |
| --- | --- | --- |
| **CD90** | ACAGTCTTGCAGGTGGCCCG | TCAGGGACCCCCACAGTGCC |
| **CD105** | CCATCTGGACTGGCACAACT | GTCTCTGTGTGGCGGTTACA |
| **CD29** | CGAATGTGAATGCCAGAGCG | AATGTCTCCCAACACGTCCC |
| **CD44** | AGAGAAAGCCAAGCGGACTC | TTCTGCAGGTTCCGTGTCTC |
| **CD166** | CCCCCAAACTTTCGCACAAG | TCTGACTCTCTGCCTCCTCC |
| **CD11b** | TGTTTCACGGAACCTCGGAG | TCCATCCATCGTGAGGTCCT |
| **CD45** | CAGGAATGAAGACCACCCCC | AGAGGATGGTGCAGTGAACG |
| **CD34** | TCACCGGGTCAGCTATCTCA | CTGCCCTGAGAGACAGTGTG |
| **CD31** | CCGGAAAGCTGACCCTGATT | CACAAGGACTGTCCGCTCTT |
| **VCAM-1** | TCCACGCTGGTCATGAATCC | TCTGGGTCCTTGGGGAAAGA |
| **vWF** | GTTTGCTGAGTGCCACAAGG | AGGTGGGCATAAGAGGCAAC |
| **Vimentin** | TCCAAGTTTGCCGACCTCTC | GACTCGTTGGTCCCCTTGAG |
| **Vinculin** | GGCTAAGAGGGAGGTGGAGA | GTTTCCTGCCACAGCCTTTG |
| **Integrin β1** | CGAATGTGAATGCCAGAGCG | AATGTCTCCCAACACGTCCC |
| **CD151** | AGCTGAGTGCAGAGCTCAAG | GGAACTCCTGCTGCAGCTTA |
| **Fibronectin** | TGCACCAACCAACCTGAAGT | CTTCATTGGCCCGGTCTTCT |
| **Integrin β2** | CCAAGAAGGTTTCGAGGGCT | CAAAGTCACACTGGCACACG |
| **LamininA5** | GGACACAGACGAGACAAGCA | CCACTGCTCCACGTTCTTCT |
| **COL4A1** | AGAGATGGCCTTGAGGGACT | TCACCCTTGAGCCGAATGTC |
| **COL1A1** | CCTGGACGCCATCAAAGTCT | AGACGTGCCTCTTGTCCTTG |
| **COL2A1** | TCCTAACACTGCCAACGTCC | GTCCAGGTAGGCAATGCTGT |
| **Β-Actin** | ATCCACGAGACCACCTTCAA | TGATCTCCTTCTGCATCCTG |
| **GAPDH** | GCTTTGCCCCGCGATCTAATGTTC | GCCAAATCCGTTCACTCCGACCTT |
| **Osteocalcin** | CAGGAGGGAGGTGTGTGAG | TGCGAGGTCTAGGCTATGC |
| **ALP** | CCAAAGGCTTCTTCTTGCTG | TGTACCCGCCAAAGGTAAAG |
| **Osteopontin** | AAGGACAGTCAGGAGACGAG | TCAATCACATTGGAATGCTC |
| **Runx2** | GAGGAACCGTTTCAGCTTACTG | CGTTAACCAATGGCACGAG |
| **Osterix** | CTCATTCCCTGGCTCAC | TGGGCAGACAGTCAGAAGAG |
| **Osteonectin** | TCCGGATCTTTCCITTGCTTTCTA | CCTTCACATCGTGGCAAGAGTTTG |
| **BSP** | GCACGCCTACTTCTATCCTC | CGGCCTCGGAGTCTT |
| **Col 1** | CCAAGAGGAGGGCCAAGAAGAAGG | GGGGCAGACGGGGCAGCACTC |
| **Aggrecan** | TTCCCTGAGGCCGAGAAC | GGGCGGTAATGGAACACAAC |
| **Sox9** | CCGGTGCGCGTCAAC | TGCAGGTGCGGGTACTGAT |
| **Col2** | CTGGAGCTCCTGGCCTCGTG | CAGATGCGCCTTTGGGACCAT |
| **AP-2** | AACCCAACCTGATCATCACTG | TCTTTCCATCCCACTTCTGC |
| **PPAR-γ** | AGGAGCAGAGCAAAGAGG | AGAGTTACTTGGTCATTCAGG |

**Table S2: Table presenting sequences of quantitative RT-PCR primers for swine genes.**

**
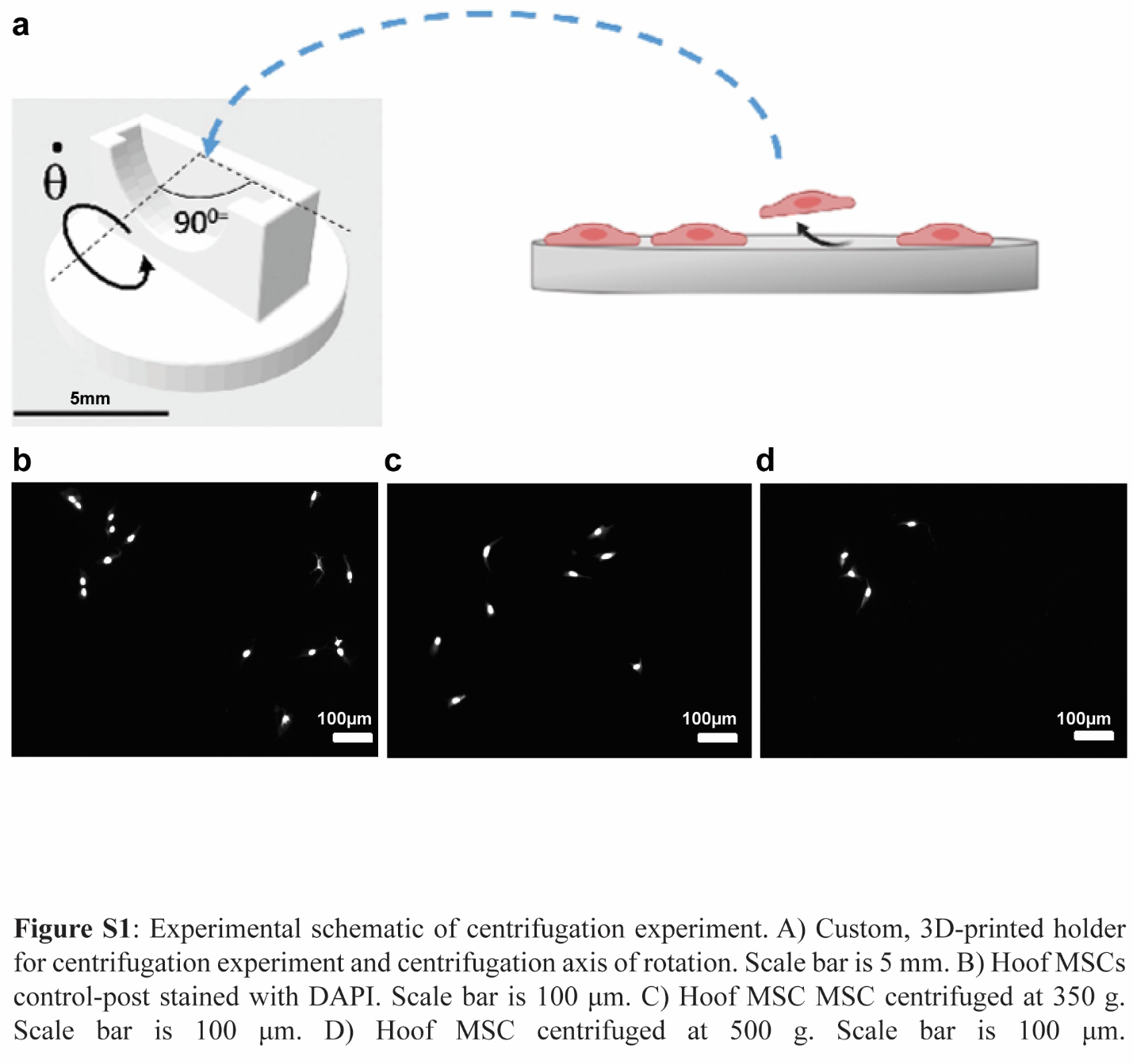
**

**Figure S1: Experimental schematic of centrifugation experiment.** A) Custom, 3D-printed holder for centrifugation experiment and centrifugation axis of rotation. Scale bar is 5 mm. B) Hoof MSCs control-post stained with DAPI. Scale bar is 100 μm. C) Hoof MSC centrifuged at 350 *g*. Scale bar is 100 μm. D) Hoof MSC centrifuged at 500 *g*. Scale bar is 100 μm.


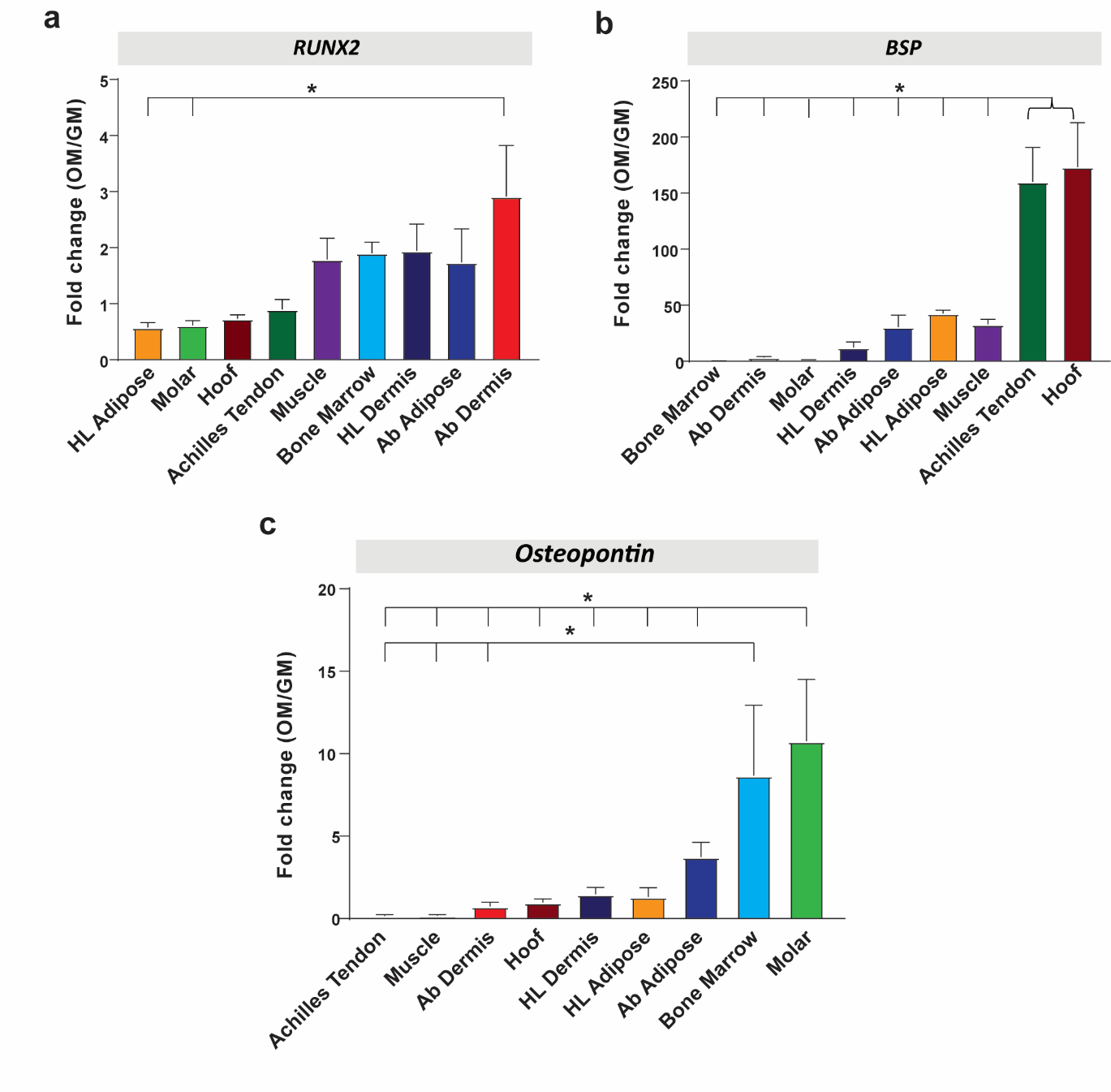


**Figure S2**. Fold change in expression of osteogenic genes A. *RUNX2*, B. *Bone sialoprotein* (*BSP*); and C. *Osteopontin* in pMSCs cultured in osteogenic media (OM) versus their levels in regular growth media (GM). Data presented as mean ± SEM (n=3; * p<0.05).

**
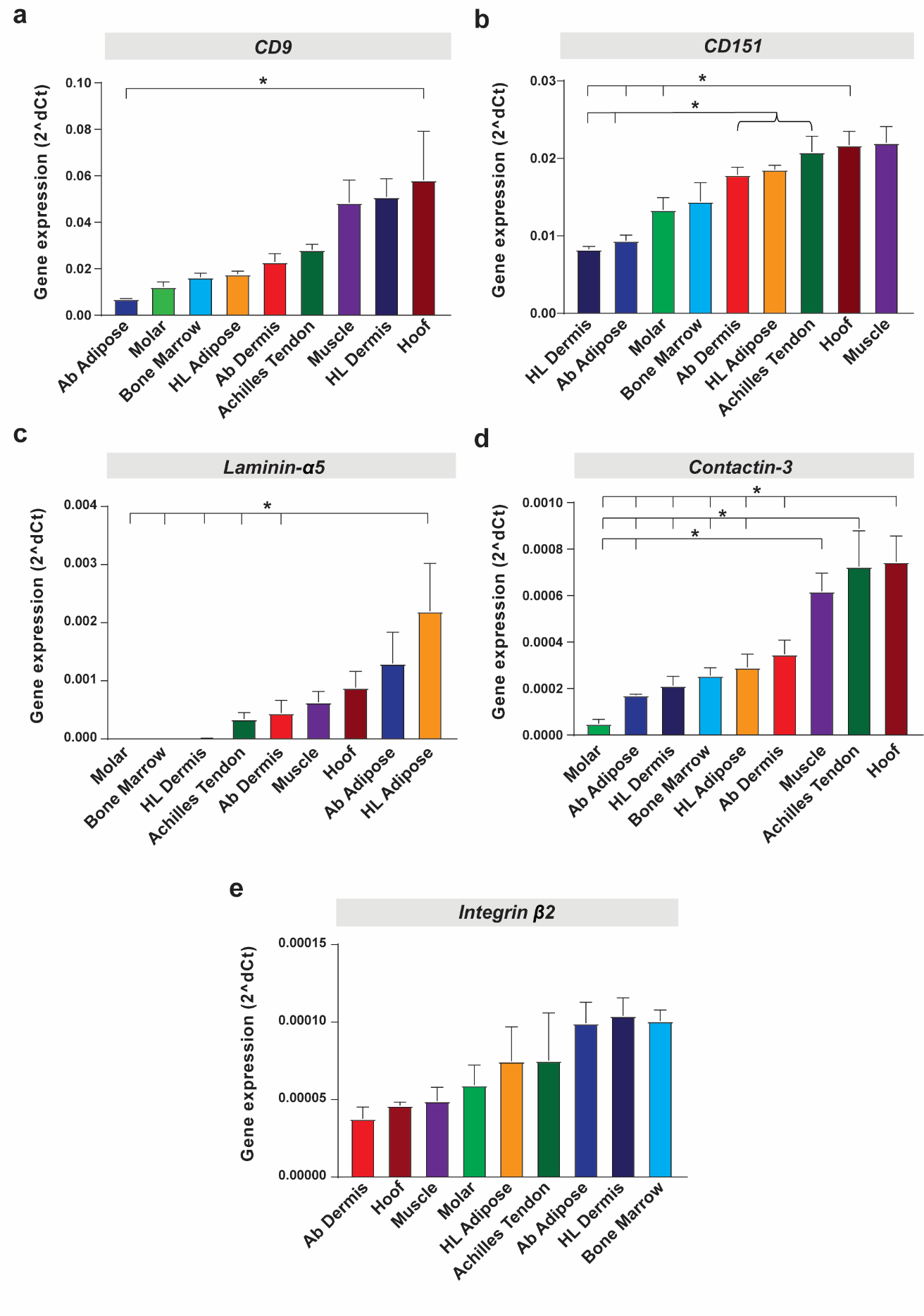
**

**Figure S3**. Gene expression profiling of cell-cell and cell-matrix genes in Passage 5 cells cultured in growth media. A. *CD9,* B. *CD151,* C. *Laminin-α5*, D. *Contactin-3*, E. *Integrin β2*. Data presented as mean ± SEM (n=3; * p<0.05).

**
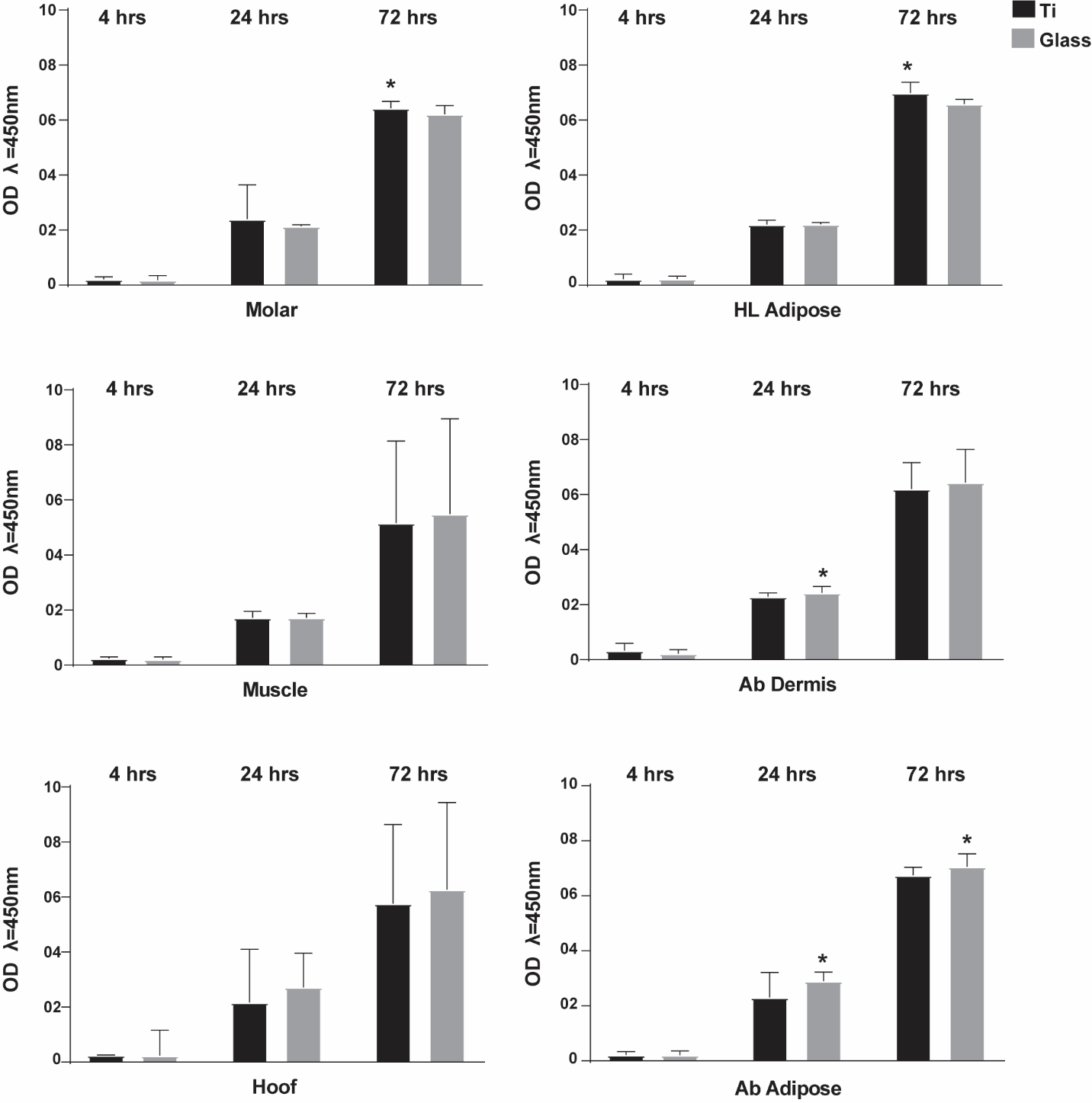
**

**Figure S4**. CCK8-based assessment of metabolic activity/cell proliferation and viability of pMSCs at 4, 24 and 72 h. (Ti: Titanium; n=3; * p<0.05 between Ti and Glass).


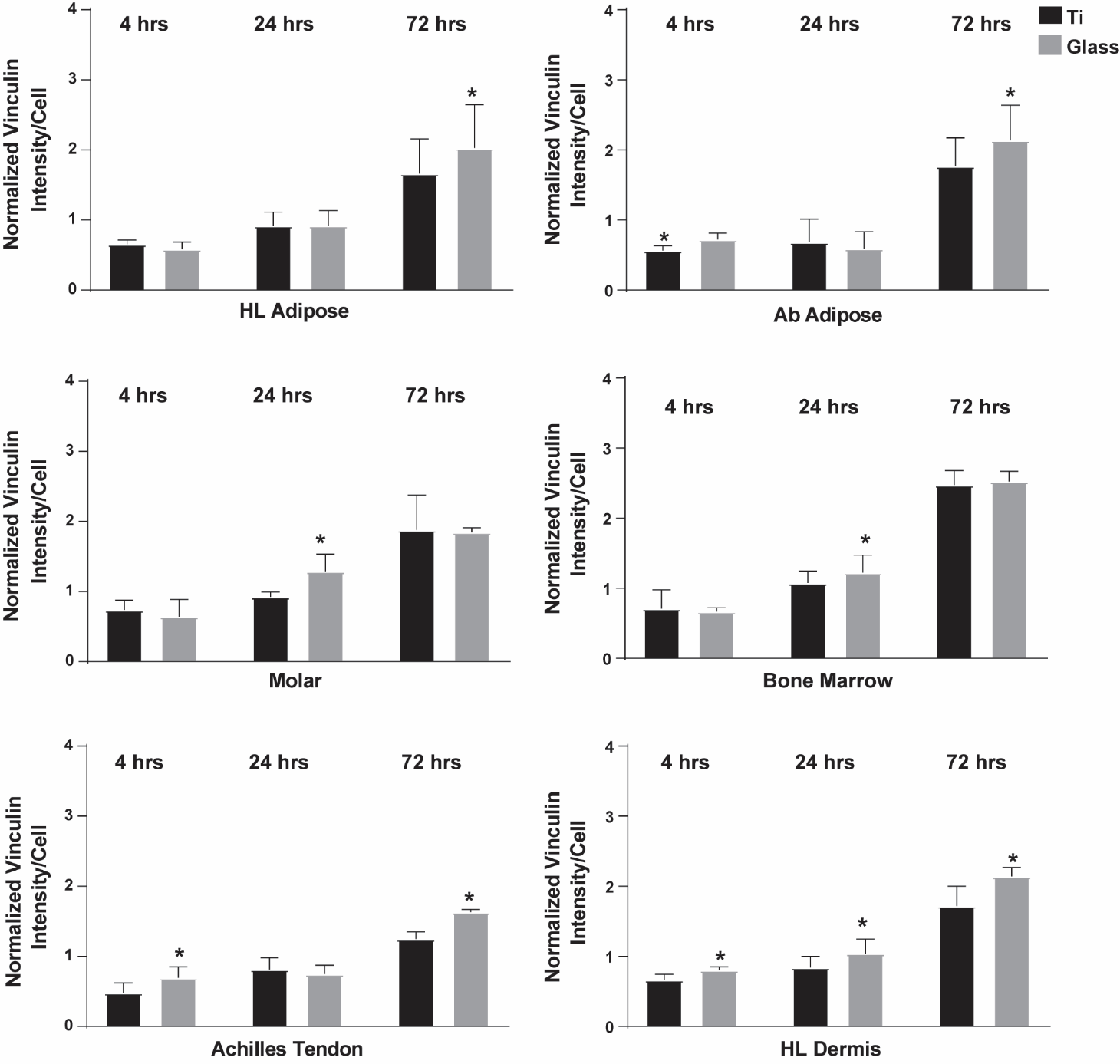


**Figure S5**. Measurement of intracellular levels of the focal adhesion protein, vinculin in pMSCs on titanium versus glass surface at 4, 24 and 72 h. (Ti: Titanium; n=3; * p<0.05 between Ti and Glass).


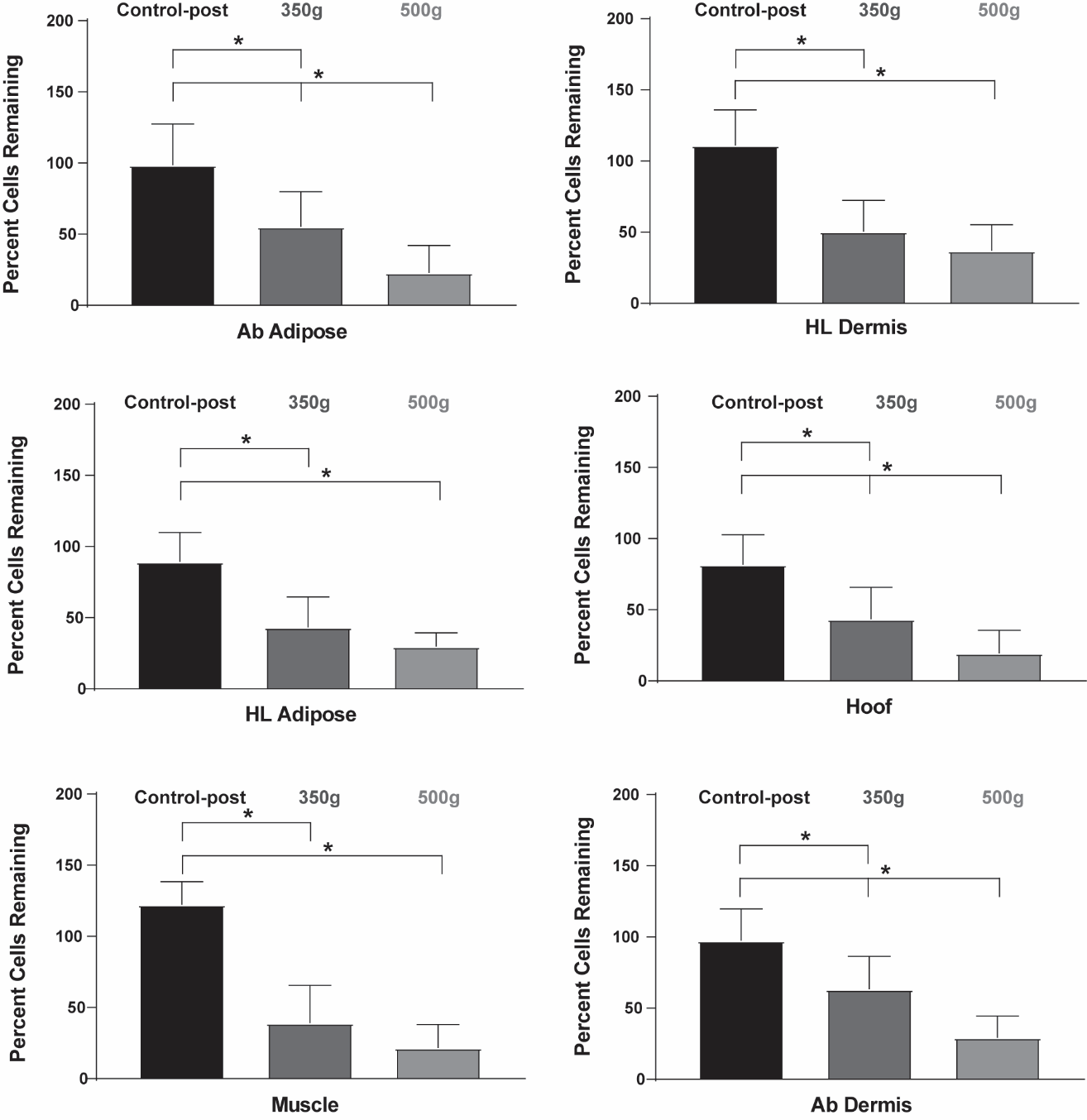


**Figure S6**. Functional assessment of adhesion potential of pMSCs to titanium. Centrifugation based functional test of adhesion of pMSCs to titanium, where control-post is the sham/mock group; 350 g and 500 g refer to the two centrifugal forces tested. (n=3; * p<0.05).
